# Atenolol, alone or in combination with PTH, has a modest effect on bone in female C57BL/6J mice

**DOI:** 10.1093/jbmrpl/ziaf087

**Published:** 2025-05-15

**Authors:** Rebecca L Fontaine, Daniel J Brooks, Deborah Barlow, Ryan J Neilson, Christine W Lary, Karen L Houseknecht, Katherine J Motyl

**Affiliations:** Center for Molecular Medicine, MaineHealth Institute for Research, MaineHealth, Scarborough, ME, 04074, United States; Graduate School of Biomedical Science and Engineering, University of Maine, Orono, ME, 04469, United States; Center for Advanced Orthopaedic Studies, Beth Israel Deaconess Medical Center, Boston, MA, 02215, United States; Department of Biomedical Sciences, Portland Laboratory for Biotechnology and Health Sciences, College of Osteopathic Medicine, University of New England, Portland, ME, 04103, United States; Center for Molecular Medicine, MaineHealth Institute for Research, MaineHealth, Scarborough, ME, 04074, United States; Department of Public Health and Health Sciences, Roux Institute at Northeastern University, Portland, ME, 04101, United States; Center for Molecular Medicine, MaineHealth Institute for Research, MaineHealth, Scarborough, ME, 04074, United States; Department of Public Health and Health Sciences, Roux Institute at Northeastern University, Portland, ME, 04101, United States; Department of Biomedical Sciences, Portland Laboratory for Biotechnology and Health Sciences, College of Osteopathic Medicine, University of New England, Portland, ME, 04103, United States; Center for Molecular Medicine, MaineHealth Institute for Research, MaineHealth, Scarborough, ME, 04074, United States; Graduate School of Biomedical Science and Engineering, University of Maine, Orono, ME, 04469, United States; Tufts University School of Medicine, Tufts University, Boston, MA, 02111, United States

**Keywords:** anabolics, bone-brain-nervous system interactions, therapeutics, diseases and disorders of/related to bone, osteoporosis, PTH/VIT D/FGF23, β-blockers

## Abstract

Atenolol is a β1-selective β-adrenergic receptor antagonist (a.k.a. β-blocker) and is under investigation in a clinical trial to prevent osteoporosis in postmenopausal women. The effects of atenolol on rodent bone are unknown, which limits research investigating mechanisms or modeling human treatment effects. However, propranolol, a non-selective β-blocker, has been widely used in rodent models. Propranolol co-treatment with intermittent truncated PTH improves a serum marker of bone formation, P1NP, while blocking the PTH-induced increase in CTX-I-MMP, a serum marker of bone resorption. To determine whether atenolol has similar properties as propranolol during co-treatment, we tested the combined effects of atenolol and PTH in female C57BL/6J mice. Atenolol exposure was confirmed in both serum and marrow at clinically relevant levels. Atenolol had little effect on femoral or L5 vertebra microarchitecture, either on its own or in combination with PTH, which improved trabecular microarchitecture as expected. However, co-treatment with PTH significantly increased P1NP levels past that of PTH alone, suggesting longer treatment may improve bone density by increasing bone formation. In summary, we found little effect of atenolol alone or in combination with PTH, which may be related to relative selectivity of atenolol for β1AR over β2AR, the predominant βAR in bone. Future studies should test whether longer term atenolol may improve microarchitectural parameters with PTH co-treatment.

## Introduction

Evidence from both pre-clinical and clinical studies has pointed to the possibility that β-blockers may be protective of bone. A short-term randomized trial found that 20 wk of atenolol, a β1-selective blocker (50 mg/d) increased BMD of the ultradistal radius and reduced the serum bone resorption marker CTx.^1^ These findings led to a clinical trial (NCT04905277), testing the effects of atenolol taken daily over 2 yr on BMD and bone turnover markers. Despite this exciting advancement in studying preventative treatments for osteoporosis, several questions remain behind atenolol’s effects on bone, including the major cell types responsible and whether the canonical mechanism of action is also replicated in bone.

The role of the sympathetic nervous system (SNS) in regulating bone homeostasis is complex. Briefly, the catecholamines norepinephrine and epinephrine are released from the adrenal glands and sympathetic nerve terminals, respectively, and bind to α- and β-adrenergic receptors (AR) on target cells. Within bone, it is well understood that the SNS reduces bone formation and leads to increased osteoclastic bone resorption through increased RANKL production, leading to low trabecular bone.^2^^,^^3^ Sympathetic activity is increased in postmenopausal women and is correlated with reduced trabecular bone and bone resorption.^4^ In mice, β2AR appears to be the major AR influencing bone.^2^ However, in humans, β1AR may be the more prominent AR controlling bone homeostasis. From a selectivity range of β-blockers tested, β1-selective blockers had more prominent effects on bone-related outcomes than non-selective β-blockers.^1^  *ADRB1* is elevated in blood of postmenopausal women and is part of a proposed 11-gene combination biomarker for postmenopausal osteoporosis, with a 94% prediction accuracy.^5^

Previous studies in rodents have found that combining the non-selective β-blocker propranolol with intermittent truncated PTH improved PTH effects on bone microarchitecture^6^^,^^7^ and fracture healing.^8^ Specifically, propranolol both promoted bone formation and prevented PTH-induced bone resorption in female C57BL/6J mice. In contrast, another study demonstrated that PTH efficacy in mice requires β2AR, indicating genetic deletion and pharmacologic inhibition do not necessarily have equivalent effects.^9^ Mechanistically, in vitro studies have found β2AR influences PTH receptor signaling in several ways. By stimulating the Gβγ G protein subunits on adenylate cyclase type 2, β2AR and PTH receptor synergistically prolonged cyclic AMP (cAMP) signaling.^10^ Knockdown of β2AR also enhanced phosphorylation of cAMP response element binding protein after PTH stimulation.^11^ In MC3T3-E1 osteoblasts, PTH treatment also suppressed *Adrb2* gene expression.^11^ Despite this, pre-treatment of MC3T3-E1 cells with propranolol significantly increased intracellular Ca^2+^ levels driven by PTH.^6^ Both β2AR and PTH type 1 receptor (PTH1R) can be manipulated to bias signaling. For example, PTH1R signaling leads to varying biological outcomes, dependent on the spatial and temporal receptor activity.^12^ PTH can also be manipulated to activate β-arrestin and not G protein signaling, leading to increased trabecular bone formation, without stimulating bone resorption.^13^ Some β-blockers, like carvedilol and nebivolol, are also β-arrestin biased ligands.^14^^,^^15^ Whether PTH and β2AR effects are due to biased signaling is unclear. Additionally, whether β1AR is required for PTH effects in bone is unknown. However, in vitro and in vivo studies in humans indicate that βAR agonists stimulate PTH secretion, and that this is specific to the β1 subtype.^16^

Although *Adrb1* is expressed less than *Adrb2* in bone, several studies have found effects of β1-selective antagonists. In a model of spontaneously hypertensive rats, atenolol inhibited tooth movement and increased alveolar bone volume by decreasing osteoclast number.^17^ The β1-selective blocker, nebivolol improved fracture healing in a rat model.^18^ Metoprolol, also a β1-selective blocker, when administered to ovariectomized rats, maintained BMD in the lumbar 4 vertebrae.^19^ Metoprolol increased osteoblast proliferation, alkaline phosphatase activity, and calcium mineralization.^19^ However, the effects on osteoclasts were not investigated. Although limited, several studies have found that osteoclasts may be directly influenced by adrenergic signaling, in addition to known osteoblast-mediated mechanisms. For example, isoproterenol increases osteoclastogenesis of BM macrophage derived and RAW264.7 cells.^20^^,^^21^ Osteoclasts also express *Adrb2* and propranolol prevents osteoclastogenesis in RAW264.7 cells^6^^,^^22^ and primary osteoclasts.^6^

Despite the excitement surrounding positive effects of propranolol on bone, it is used much less often clinically than the more β1-selective βAR antagonists, such as atenolol, which is being tested in a clinical trial. However, whether atenolol would act in a similar manner to propranolol and improve bone microarchitecture was unknown. Our major goals were to determine if atenolol was protective of bone in a mouse model and to test whether atenolol influences PTH efficacy. Briefly, we found that atenolol had little effect on bone in either the femur or the L5 vertebrae when dosed at clinically relevant levels. Combination of atenolol with PTH did not improve bone microarchitecture beyond that of PTH alone, despite an elevation of P1NP levels during PTH treatment. Future studies should test whether longer term atenolol and PTH treatment, or atenolol treatment in other high-resorptive states, like ovariectomy, would improve bone microarchitecture.

## Materials and methods

### Mice and drug treatment

All procedures in this study were approved by the MaineHealth Institute for Research Institutional Animal Care and Use Committee. All mice were housed in a barrier animal facility at the MaineHealth Institute for Research, an Assessment and Accreditation of Laboratory Animal Care accredited facility. Mice were provided with water and regular chow (Teklad global 18% protein diet #2918, Envigo) ad libitum and kept on a 14-h light/10-h dark cycle at room temperature (22 °C). For atenolol exposure studies, atenolol exposure (10 mg/kg) was first determined after oral gavage in 8-wk-old female C57BL/6*J* mice from the Jackson Laboratory (Strain #000664). Tissues were collected either 30 min, 1 h, or 2 h after atenolol administration or 1 h after vehicle administration. Of the mice utilized for the 4-wk treatment of atenolol and PTH, 5 mice had to be euthanized early and excluded from the study. Three of these had dermatitis or other skin issues and 2 experienced stress with oral gavage.

For co-treatment studies, female C57BL/6J mice were obtained at 13 wk of age from the Jackson Laboratory. Mice were allowed to acclimate until 16 wk of age. Mice underwent baseline DXA and were then randomly assigned to 1 of the 4 treatment groups: (1) vehicle and vehicle (*N* = 7); (2) vehicle and PTH (*N* = 8); (3) atenolol and vehicle (*N* = 7); or (4) atenolol and PTH (*N* = 8). Oral gavage was used to administer vehicle and atenolol and subcutaneous injection to administer vehicle and PTH. At 20 wk of age, tissues were collected 1 h after treatment and either fixed in 70% ethanol or snap-frozen in liquid nitrogen and stored at −80 °C. Previous BV/TV% data from propranolol and PTH treated mice was utilized to determine an *n* = 4 would be needed to detect a 20%-30% difference in the means with 80% power and *p* < .05.

### PTH and atenolol storage and preparation

PTH (4011476, previously H-1660, Bachem) was aliquoted and stored in glass vials as a 10^−4^ M stock in 4 mM HCl supplemented with 0.1% bovine serum albumin, under argon gas atmosphere, and stored at −80 °C. PTH was thawed and diluted in 0.9% saline on ice immediately prior to injection (80 μg/kg). Atenolol (A7655, Sigma-Aldrich) was dissolved in autoclaved diH_2_O and delivered at 10 mg/kg.

### Liquid chromatography with tandem mass spectrometry

To determine atenolol levels in the serum and marrow, liquid chromatography with tandem mass spectrometry (LC-MS/MS) was used. Marrow samples were diluted 1:1 with 50 mM potassium phosphate buffer (pH 7.4) and homogenized until uniform. Calibration standards were prepared from 10 000 to 1.00 nM. Twenty microliters blank serum, calibration standard, or sample were added to a microcentrifuge, mixed with 100 μL acetonitrile for 2 min, and centrifuged for 5 min at 18407 g. One-hundred microliters of the supernatant was added to a 96 well plate and 2.0 μL were injected for LC-MS/MS using an Agilent 1200 system. Chromatographic separation was performed using Zorbax SB C18 2.1 × 50 mm 2.7 μL column via a gradient using 0.1% formic acid in water (A) and 0.1% formic acid in acetonitrile (B). For single-dosing measurements, gradient elution was 95% A from 0.0 to 3.0 min, 20% A from 3.0 to 3.1 min, and back to 95% A from 3.1 to 5.0 min. Samples from the 4-wk treatment were separated using a Phenomenex Synergi 4 μ Polar-RP 80 Å 150 × 4.6 mm column. The same gradient was used as described above. The gradient elution was 95% A from 0.0 to 4.0 min, 20% A from 4.0 to 5.0 min, and 95% from 5.1 to 8.0 min. The flow rate was 0.75 mL/min and column temperature 30 °C in both studies. Detection of atenolol was obtained using an Agilent 6460 triple quadrupole mass spectrometer, monitoring the transition from 267.17 → 145.0 with a fragmentor of 95 V and collision energy of 33 V. The retention time of atenolol with Zorbax column separation was 1.61 and 4.38 min with Phenomenex column separation.

### Dual-energy X-ray absorptiometry

Baseline and endpoint DXA scans were performed using the PIXImus dual-energy X-ray densitometer (GE-Lunar). Daily calibration was performed using a manufacturer provided phantom mouse. Fat-free mass, fat mass, BMD, and bone mineral content were measured. A full body scan was obtained once anesthetized and placed ventral side down with the limbs and tail away from the body. X-ray energy absorptiometry data was processed, excluding the head, using manufacturer supplied software (Lunar PIXImus 2, version 2.1).

### Micro-computed tomography

The microarchitecture of the fifth lumbar (L5) vertebral body and the femur were analyzed using a high-resolution desktop micro-tomographic imaging system (μCT40, Scanco Medical AG). Scans were acquired using a 10 μm^3^ isotropic voxel size, 70 kVp peak X-ray tube intensity, 114 mA X-ray tube current, 200 ms integration time, and subjected to Gaussian filtration and segmentation. Within the femur, 2 regions were analyzed in the distal metaphysis including a primary spongiosa region of interest that started at the peak of the distal growth plate and extended proximally for 500 μm, and a secondary spongiosa region of interest that started immediately superior to the primary spongiosa region of interest and extended 1500 μm proximally. The region of interest within the L5 vertebrae was a 750 μm diameter × 2 mm long cylindrical region centralized on the vertebral body. A threshold of 400 mgHA/cm^3^ was used to segment bone from soft tissue for trabecular analyses. Cortical bone was analyzed at the femoral mid-diaphysis and segmented with the mineral density of 700 mgHA/cm^3^. All analyses were performed using the Scanco Evaluation software and cortical and trabecular bone architecture were analyzed using the Scanco mid-shaft evaluation and trabecular bone morphometry scripts, respectively.

### Bone turnover markers

Serum concentrations of amino-terminal P1NP and cross-linked C-telopeptide (CTX-I-MMP)^23^ were measured with enzyme immunoassays (EIA, Immunodiagnostic Systems). Assays were performed following manufacturer’s instructions and all measurements were performed in duplicate.

### RNA isolation and real-time PCR (qPCR)

Total RNA was isolated from whole tibia using the standard TRIZOL (Sigma) method. cDNA was synthesized using the High Capacity cDNA Reverse Transcriptase Kit (Applied Biosystems) and RNase-free DNase (Roche) according to manufacturer’s instructions. mRNA expression analysis was performed using an iQ SYBR Green Supermix Assay with an Opus thermal cycler and detection system (Bio-Rad). Hypoxanthine guanine phosphoribosyl transferase (*Hprt*) was used as an internal standard control gene. Primer sequences are listed in Table S4, and if possible, primers were designed to span introns. Specificity of primer amplification was verified by performing a melt curve analysis.

### Statistical analysis

GraphPad Prism 10.1.2 XML Project software was used to perform statistical analyses. Statistical analysis on gene expression data was performed on delta Ct values while plots demonstrate fold change. Data were analyzed using a 2-way ANOVA, followed by Tukey multiple comparison post hoc test, where appropriate. Statistically significant values were determined to be *p* < .05 and all data are expressed as the mean ± SD. Graphical data is presented as boxplots with individual points plotted as closed circles. Boxes represent the 25th to 75th percentiles, with the horizontal line indicating the median.

## Results

Prior to initiating the 4-wk treatment, we performed a single-dose pharmacokinetic study to determine concentrations of atenolol achieved in serum and marrow following our dosing regimen (Table S1). After 30 min, 1 h, and 2 h, atenolol concentrations in the serum were comparable to those achieved clinically.^24^^,^^25^ During our 4-wk treatment study, atenolol was present in serum of mice treated with atenolol or atenolol and PTH (but levels were undetectable in vehicle and PTH treated mice). Within the marrow, atenolol levels were on average 5.5 times higher than in the serum (Figure S1).

**Figure 1:**
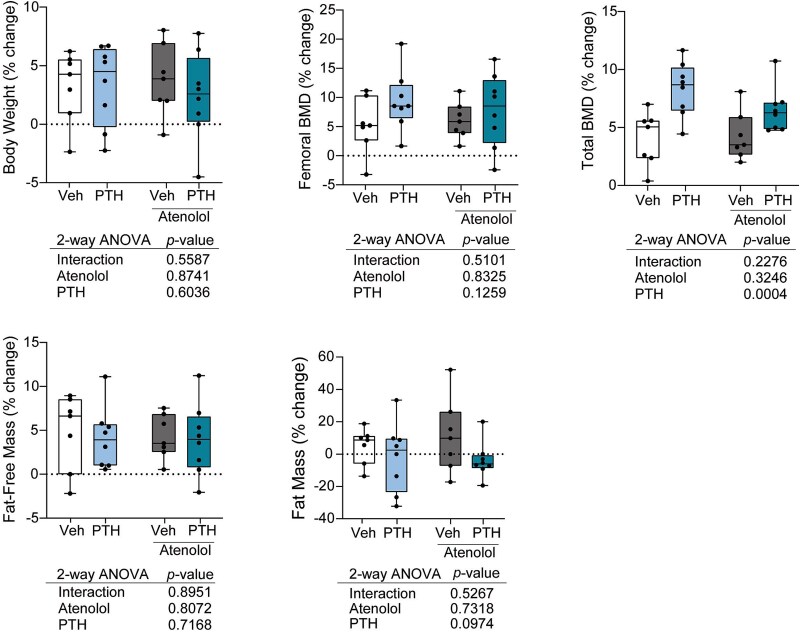
Neither atenolol or PTH influence body mass or composition. Dual-energy X-ray absorptiometry was performed at baseline and endpoint. Female C57BL/6J mice were treated with vehicle, 80 μg/kg PTH, 10 mg/kg atenolol, or PTH and atenolol for 4 wk from 16 to 20 wk of age. Percent change between baseline and endpoint was calculated for each mouse. *N* = 7-8/group. Individual points are plotted as closed circles. Boxes represent the 25th to 75th percentiles, with the horizontal line indicating the median. Data was analyzed by 2-way ANOVA, with main effects (interaction, atenolol, PTH) *p*-values shown below the plots.

Baseline DXA parameters were largely not different between groups, although body mass was different in mice assigned to receive atenolol (Table S2). As expected, PTH increased total areal BMD but did not influence body composition. Atenolol did not influence any change in body composition or bone parameters (Figure 1, Table S3).

In the distal femur, and as expected, PTH had an anabolic effect on trabecular bone in both the primary and secondary spongiosa. However, atenolol had no effect in the secondary spongiosa alone or in combination with PTH treatment. PTH increased several cortical parameters in the femur mid-diaphysis including cortical area (Ct.Ar), total area (Tt.Ar), cortical bone area fraction (Ct.Ar/Tt.Ar), cortical tissue mineral density (Ct.TMD), cortical thickness (Ct.Th), and moments of inertias (pMOI, *I*_min_, and *I*_max_). In the primary spongiosa, total volume (TV) was slightly elevated in atenolol treated mice (main effect, Table 1), but there were no differences in bone volume (BV) or bone volume fraction (BV/TV). Atenolol effects within the cortical bone at the femur mid-diaphysis were limited to increased medullary area (Table 2). Atenolol did not influence the effects of PTH treatment on cortical bone.

**Table 1:** Trabecular microarchitecture of the distal femur of mice treated with PTH and/or atenolol.

	**Vehicle** **(*N* = 7)**	**PTH** **(*N* = 8)**	**Atenolol** **(*N* = 7)**	**PTH + Atenolol** **(*N* = 8)**	**2-way ANOVA *p*-values**		
					**PTH**	**Atenolol**	**Interaction**
**Distal femur secondary spongiosa**							
**Tb.BV/TV (%)**	7.28 ± 1.7	10.18 ± 1.4	7.02 ± 1.4	9.91 ± 1.8	**<.0001**	.6434	.9865
**Tb.BMD (mgHA/cm**^**3**^**)**	177 ± 16	203 ± 11	179 ± 17	207 ± 24	**.0003**	.6831	.9331
**Conn.D (1/mm**^**3**^**)**	49 ± 18	88 ± 13	57 ± 18	90 ± 15	**<.0001**	.4289	.5871
**SMI**	3.02 ± 0.23	2.61 ± 0.10	2.96 ± 0.21	2.59 ± 0.13	**<.0001**	.5138	.7195
**Tb.N (1/mm)**	3.64 ± 0.25	3.80 ± 0.17	3.63 ± 0.21	3.85 ± 0.37	.0603	.8208	.7644
**Tb.Th (mm)**	0.046 ± 0.003	0.052 ± 0.004	0.045 ± 0.003	0.051 ± 0.005	**.0002**	.3122	.8589
**Tb.Sp (mm)**	0.276 ± 0.022	0.264 ± 0.011	0.276 ± 0.016	0.262 ± 0.021	.0544	.8945	.9114
**Distal femur primary spongiosa**							
**BV/TV (%)**	45.3 ± 4.1	62.5 ± 5.6	44.4 ± 2.9	59.5 ± 5.6	**<.0001**	.2662	.5613
**BV (mm**^**3**^**)**	0.734 ± 0.059	1.031 ± 0.120	0.742 ± 0.057	1.017 ± 0.100	**<.0001**	.9359	.7338
**TV (mm**^**3**^**)**	1.62 ± 0.04	1.65 ± 0.05	1.67 ± 0.08	1.71 ± 0.06	.1763	**.0165**	.8393
**BMD (mgHA/cm**^**3**^**)**	430 ± 28	530 ± 30	424 ± 27	510 ± 37	**<.0001**	.2680	.5525
**TMD (mgHA/cm**^**3**^**)**	842 ± 8	813 ± 14	840 ± 11	809 ± 12	**<.0001**	.4984	.7302

Data presented as mean ± SD. Abbreviations: BV, bone volume; Conn.D, connectivity density; *N*, number; PTH, parathyroid hormone; SMI, structure model index; Sp, separation; Tb, trabecular; Th, thickness; TMD, tissue mineral density; TV, total volume. Bold values highlight *p* < 0.05.

**Table 2:** Cortical microarchitecture of femur midshaft of mice treated with PTH and/or atenolol.

	**Vehicle** **(*N* = 7)**	**PTH** **(*N* = 8)**	**Atenolol** **(*N* = 7)**	**PTH + Atenolol** **(*N* = 8)**	**2-way ANOVA *p*-values**		
					**PTH**	**Atenolol**	**Interaction**
**Ct.Ar (mm** ^**2**^ **)**	0.826 ± 0.056	0.916 ± 0.050	0.841 ± 0.041	0.917 ± 0.054	**.0001**	.6517	.7355	
**Ma.Ar (mm** ^**2**^ **)**	0.872 ± 0.014	0.910 ± 0.061	0.935 ± 0.041	0.922 ± 0.039	.4203	**.0259**	.1208	
**Tt.Ar (mm** ^**2**^ **)**	1.70 ± 0.052	1.83 ± 0.104	1.78 ± 0.061	1.84 ± 0.069	**.0018**	.1112	.2591	
**Ct.Ar/Tt.Ar (%)**	48.6 ± 1.9	50.2 ± 1.1	47.4 ± 1.5	49.9 ± 1.8	**.0016**	.1928	.4216	
**Ct.Th (mm)**	0.196 ± 0.012	0.210 ± 0.008	0.194 ± 0.008	0.210 ± 0.011	**.0003**	.7933	.7665	
**Ct.TMD (mgHA/cm** ^**3**^ **)**	1195 ± 11	1210 ± 13	1195 ± 9	1209 ± 9	**.0007**	.9397	.8815	
**Ct.Por (%)**	1.06 ± 0.15	1.13 ± 0.22	1.07 ± 0.15	1.15 ± 0.25	.3207	.8664	.9731	
**pMOI (mm** ^**4**^ **)**	0.359 ± 0.033	0.427 ± 0.048	0.386 ± 0.035	0.429 ± 0.041	**.0009**	.3350	.4176	
***I***_**max**_ **(mm**^**4**^**)**	0.243 ± 0.026	0.292 ± 0.024	0.261 ± 0.033	0.292 ± 0.033	**.0024**	.4663	.4663	
***I***_**min**_ **(mm**^**4**^**)**	0.116 ± 0.009	0.135 ± 0.014	0.125 ± 0.008	0.137 ± 0.010	**.0003**	.1350	.3869	

Data presented as mean ± SD. Abbreviations: Ar, area; Ct, cortical; *I*_max_, maximum moment of inertia; *I*_min_, minimum moment of inertia; Ma, medullary; Por, porosity; pMOI, polar moment of inertia; Th, thickness; TMD, tissue mineral density; Tt, total. Bold values highlight *p* < 0.05.

**Figure 2:**
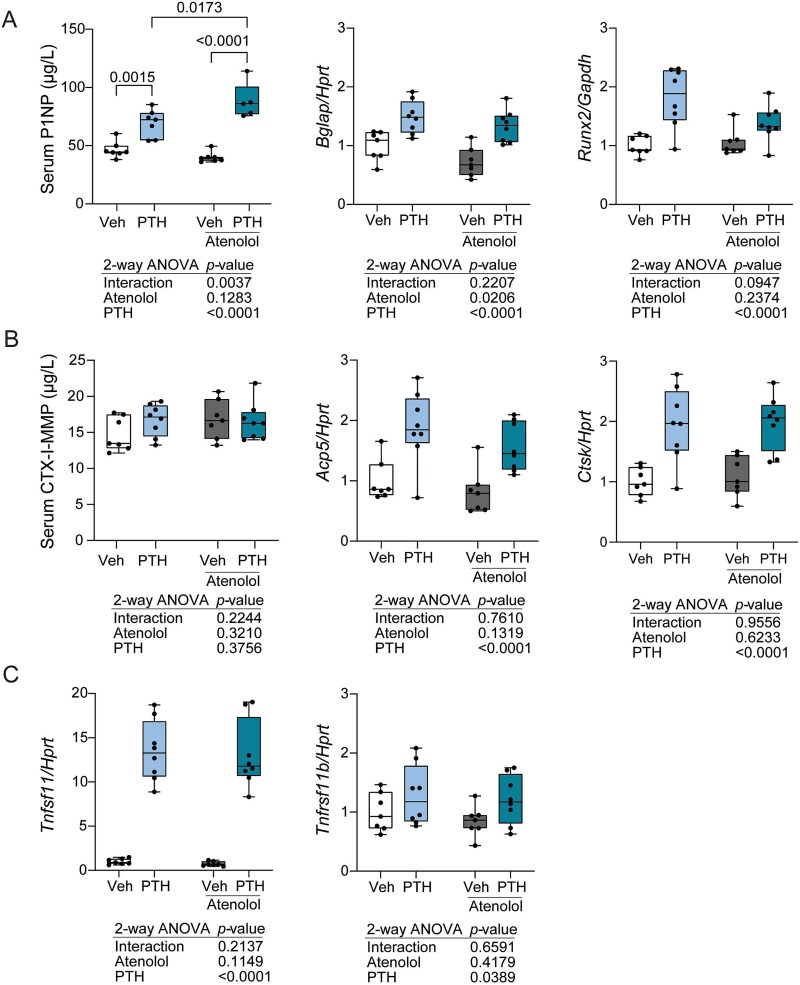
Atenolol increased circulating P1NP, but did not alter CTX-I-MMP, during PTH treatment. Female C57BL/6J mice were treated with vehicle, 80 μg/kg PTH, 10 mg/kg atenolol, or PTH and atenolol for 4 wk from 16 to 20 wk of age. Endpoint serum was collected for bone turnover marker measurements and whole tibia for gene expression. (A) Bone formation marker P1NP was measured in serum and markers *Bglap* and *Runx2* were measured in whole tibia. (B) Bone resorption marker CTX-I-MMP was measured in serum and markers *Ctsk* and *Acp5* were measured in whole tibia. (C) Gene expression markers of *Tnfsf11* and *Tnfrsf11b*, genes encoding RANKL and OPG respectively, measured in whole tibia. Gene expression was normalized to the non-modulated housekeeping gene, *Hprt*; gene expression *N* = 7-8/group. Serum EIA *N* = 5-8/group. Individual points are plotted as closed circles. Boxes represent the 25th to 75th percentiles, with the horizontal line indicating the median. Data was analyzed by 2-way ANOVA, with main effects (interaction, atenolol, PTH) *p*-values shown below the plots. Tukey’s post hoc test was performed only when there was a significant (*p* < .05) interaction and resulting pairwise *p*-values are shown on the brackets above the boxplots.

**Figure 3:**
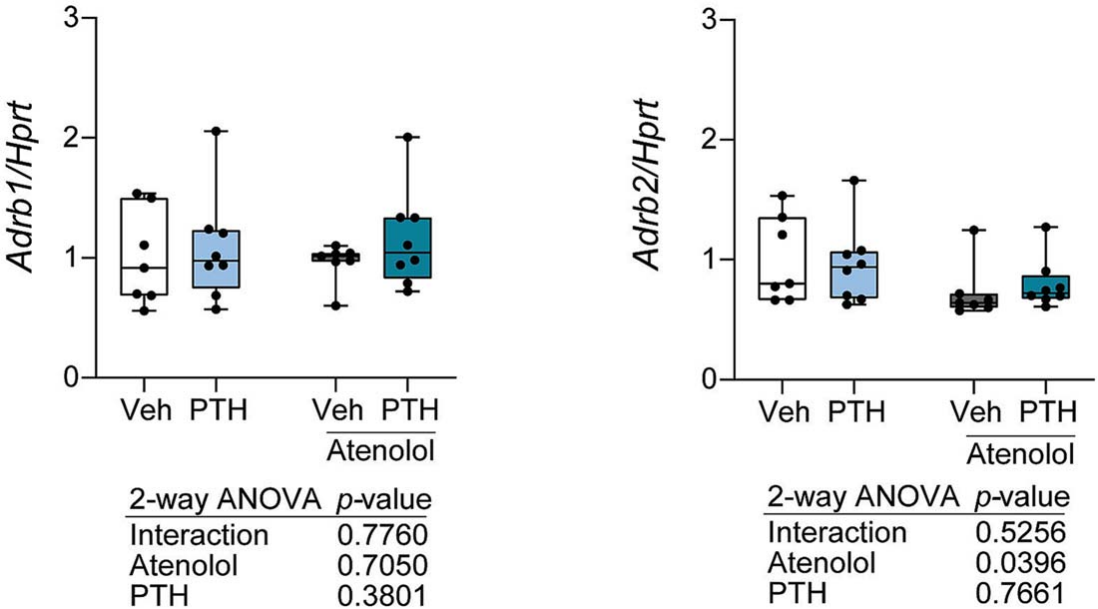
Atenolol reduces expression of *Adrb2,* but not *Adrb1*. Female C57BL/6J mice were treated with vehicle, 80 μg/kg PTH, 10 mg/kg atenolol, or PTH and atenolol for 4 wk from 16 to 20 wk of age. Gene expression of *Adrb1* (average Ct = 26.6) and *Adrb2* (average Ct = 21.7) measured in whole tibia. Gene expression was normalized to the non-modulated housekeeping gene, *Hprt*; gene expression *N* = 7-8/group. Individual points are plotted as closed circles. Boxes represent the 25th to 75th percentiles, with the horizontal line indicating the median. Data was analyzed by 2-way ANOVA, with main effects (interaction, atenolol, PTH) *p*-values shown below the plots.

Despite observing little change in femoral microarchitecture with atenolol treatment, we measured serum and whole tibia bone turnover markers to determine if atenolol influenced turnover. Consistent with the known anabolic effects, PTH increased serum P1NP, tibia *Bglap*, and tibia *Runx2*. However, *Bglap* was the only formation marker influenced by atenolol treatment alone, and it was decreased. Atenolol did influence the PTH effect on P1NP (*p* = .0037 for interaction; Figure 2A). Specifically, P1NP was significantly elevated in the PTH + atenolol group, compared to PTH alone (*p* = .0173). Regarding resorption markers, we detected a significant effect of PTH on *Ctsk* and *Acp5*, but no PTH-mediated increase in CTX-I-MMP (Figure 2B). Atenolol did not influence these markers, and we did not find any interaction effect of PTH and atenolol on CTX-I-MMP, *Ctsk*, or *Acp5* levels. As expected, we found increased expression of *Tnfsf11* (*Rankl*) and *Tnfrsf11b* (*Opg*) with PTH treatment (Figure 2C). Atenolol did not modulate *Tnfsf11* (*Rankl*) or *Tnfrsf11b* (*Opg*), nor did atenolol influence the PTH effect on these parameters (Figure 2C). We found no PTH effect on the expression of *Adrb1* or *Adrb2*. Despite no atenolol effect on *Adrb1*, atenolol lowered the expression of *Adrb2* in whole tibia, but did not cause an interaction effect (Figure 3).

**Figure 4:**
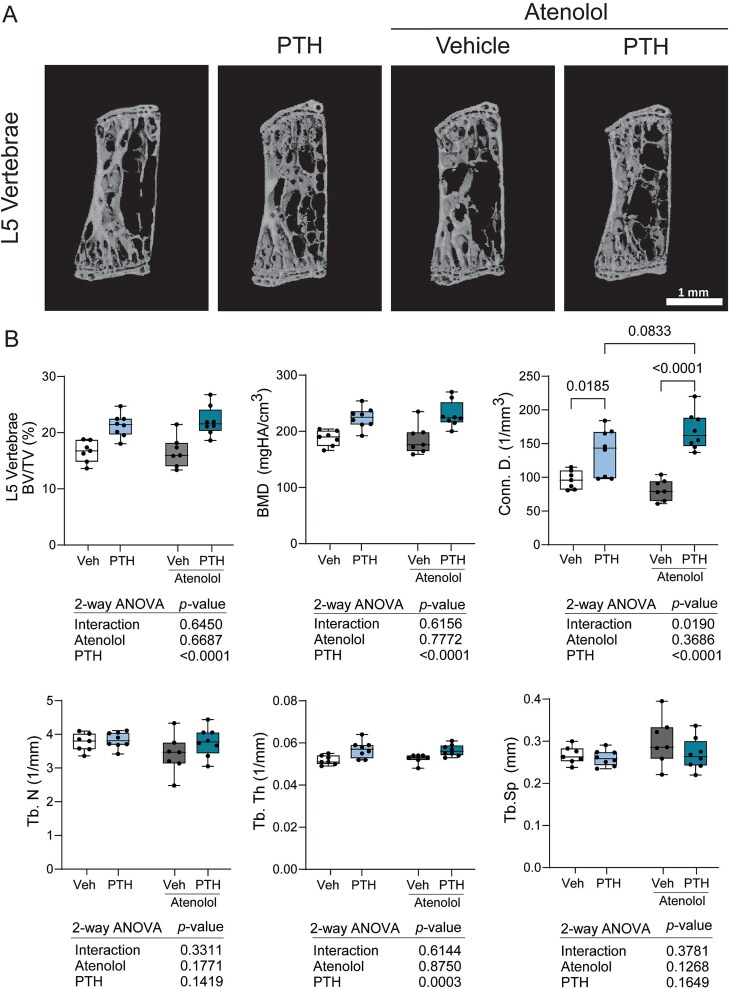
Atenolol treatment alone or in the presence of PTH treatment has limited positive effects on L5 trabecular microarchitecture. Mice were treated with vehicle, 80 μg/kg PTH, 10 mg/kg atenolol, or PTH and atenolol for 4 wk from 16 to 20 wk of age. (A) Representative images of the L5 vertebral body. (B) Trabecular bone volume fraction (Tb.BV/TV), BMD (Tb.BMD), connectivity density (Conn.D.), trabecular number (Tb.N), thickness (Tb.Th), and separation (Tb.Sp). *N* = 7-8/group. Individual points are plotted as closed circles. Boxes represent the 25th to 75th percentiles, with the horizontal line indicating the median. Data was analyzed by 2-way ANOVA, with main effects (interaction, atenolol, PTH) *p*-values shown below the plots. Tukey’s post hoc test was performed only when there was a significant (*p* < .05) interaction and resulting pairwise *p*-values are shown on the brackets above the boxplots.

Because we observed an increase in circulating P1NP levels with atenolol and PTH co-treatment, we analyzed the L5 vertebrae with μCT to determine if atenolol may cause site-specific effects. We observed expected increases with PTH treatment (Tb.BV/TV, Tb.BMD, connectivity density, and Tb.Th) (Figure 4). Atenolol had no main effect on vertebral microarchitecture, and no interaction effect on the majority of parameters. However, connectivity density was modulated as a result of a significant interaction effect, with atenolol and PTH co-treatment tending to increase connectivity density (*p* = .0833) compared to PTH alone (Figure 4).

## Discussion

Here we report the first study, to our knowledge, testing the effects of atenolol on bone in a mouse model. We found atenolol largely did not influence bone microarchitecture. We also found little influence of atenolol treatment on PTH-induced anabolism. Despite no change in bone density or bone volume fraction, atenolol promoted P1NP levels within the PTH-treated group, compared to PTH alone. Thus, we cannot exclude that a longer duration treatment of atenolol and PTH would have additive anabolic effects.

Previous studies administered atenolol at a dose of 10 mg/kg p.o. for 4 wk in mice and observed significantly reduced heart rate.^26^ In humans, the peak plasma concentration of atenolol after oral administration is reported to range from 290 ng/mL in young healthy patients to 380-600 ng/mL in hypertensive patients.^24^^,^^25^ Our studies achieved plasma atenolol exposure within range consistent with clinical relevance. On average, atenolol levels in our study were 311.6 ng/mL in the serum and 1752.4 ng/g in the marrow (Figure S1). Several preclinical models with propranolol treatment, including our own, have administered drug through the drinking water. While dosing is more frequent in the drinking water, drug exposure is more variable due to differences in the amount of water consumed than through controlled administration utilizing oral gavage. Although our dosing was clinically-relevant and well-controlled, we cannot exclude that drinking water administration of atenolol would be beneficial.

Atenolol treatment led to minimal effects including increased total volume in the primary spongiosa (Table 1) and increased medullary area in the cortical microarchitecture of the femur midshaft (Table 2). We found improvements in bone parameters with PTH treatment, as expected. However, co-treatment with atenolol and PTH largely yielded similar results to that of PTH alone. PTH treatment typically increases both serum P1NP and CTX-I-MMP levels. Our study found significantly increased P1NP levels, however, CTX-I-MMP levels only tended to be increased. Within the vehicle treated group, some mice had higher CTX-I-MMP levels which may be due to stress from both daily oral gavage and subcutaneous injections. Gene expression of bone resorption markers *Acp5* and *Ctsk* were significantly increased with PTH in the whole tibia, despite no change in circulating CTX-I-MMP (Figure 2B). We also found the expected increase in *Tnfsf11* gene expression in the whole tibia with PTH treatment (Figure 2C). Interestingly, serum P1NP levels were further increased with atenolol during PTH treatment (Figure 2A). Whether these changes were reflective at a cellular level and driven by increased osteoblast function is unknown. Yet, that largely had no effect on bone volume or mineral density. The only interaction effect found was increased connective density in the L5 vertebrae (Figure 4). However, this could not be explained by changes in other trabecular parameters.

This work further supports differences in the importance of β1- vs β2AR between species. Previous work has established differences in β1- and β2AR expression between species. In the thalamus and hippocampus, rats and guinea pigs have stark differences in receptor population distribution and number.^27^ Additionally, while β1AR predominates in rat hippocampus, it is β2AR that is more dominant in human hippocampus.^28^ The opposition in β1- and β2AR expression may also translate to bone phenotypes between species. Both pharmacological and genetic models have identified that a loss of β2AR leads to high bone mass in murine models.^3^^,^^29^ However, in human studies evidence suggests β1AR is the most important receptor.^1^ Several human studies found β1AR-selective antagonists improve BMD and reduce fracture risk,^1^^,^^30–32^ but both human and rodent studies examining the effects of β1AR in bone are limited. When considering the selectivity of βAR antagonists, atenolol is only mildly selective, with a selectivity ratio of only 4.7 when comparing β1 and β2AR.^33^ Additionally, atenolol has binding affinity at β2AR, after correcting for membrane binding, similar to salbutamol and salmeterol (β2AR-selective agonists).^34^ In our own study, atenolol did not affect gene expression levels of *Adrb1* but significantly decreased gene expression levels of *Adrb2* (Figure 3D). Whether these changes are reflective at the protein level or alter downstream signaling pathways is unknown.

In contrast to our findings with atenolol, metoprolol improves bone mass in an ovariectomized rodent model^19^ and nebivolol improves fracture healing in a rat model.^18^ Although we do not know whether atenolol influences fracture healing, differential mechanisms may explain apparent contrasting effects. Atenolol, metoprolol, and nebivolol are all β1-selective, but with different selectivity ratios. Metoprolol has an even lower selectivity ratio than atenolol (2.3 for β1 vs β2AR). Nebivolol has a much higher selectivity (selectivity ratio 321 between β1 and β2AR).^35^ The opposite findings of nebivolol are suggested to be caused by vasodilator and anti-oxidative effects via the nitric oxide pathway.^18^ Atenolol’s influence on nitric oxide has been reported to have a dual effect, dependent on the dose. At a low dose (50 ng/mL) atenolol increases nitric oxide, however at a high dose (250 ng/mL), decreases nitric oxide.^36^ Another human study found nebivolol increased free radicals, while atenolol did not.^35^ Atenolol also modulates metabolic profile, while nebivolol does not, which may also explain some of the differences.^37^ Furthermore, nebivolol also acts as a G protein-coupled receptor kinase/β-arrestin-biased agonist,^15^ and therefore signals somewhat disparately from atenolol.

In conclusion, we have shown that moderate duration administration of atenolol has little effect on bone density in female C57BL/6J mice. However, we cannot rule out the possibility that a longer duration of treatment may lead to improved BMD or whether atenolol has an effect in other stimulated states, such as after ovariectomy. Despite these possibilities, our model achieved clinically relevant drug exposures and a treatment length in which other β-blockers cause an effect. Further, we have identified that co-treatment with atenolol slightly enhances the anabolic effect of PTH treatment, but these changes do not significantly impact bone density or microarchitecture in the current treatment paradigm. An ongoing long-term clinical trial (NCT04905277) will demonstrate the effect of atenolol on bone in humans. Further work is needed to identify whether differences in adrenergic signaling between rodents and humans are due to adrenergic receptor population differences, signaling mechanisms, or other factors. Future work should also identify the extent to which atenolol impacts each cell type and its mechanism of action to aid in accurate interpretation of clinical studies and extend our understanding of regulation of bone via the SNS.

## Supplementary Material

Fontaine_Supplementary_Material_4_22_2025_ziaf087

## Data Availability

Data is available upon reasonable request.
