# Supplementary material for: Atenolol, alone or in combination with PTH, has a modest effect on bone in female C57BL/6J mice
Source: JBMR Plus. 2025 May 15;9(7):ziaf087. doi: 10.1093/jbmrpl/ziaf087 (PMC12161496; doi:10.1093/jbmrpl/ziaf087)
Supplement: Fontaine_Supplementary_Material_4_22_2025_ziaf087 [file fontaine_supplementary_material_4_22_2025_ziaf087.docx]

**Supplementary Material**

**
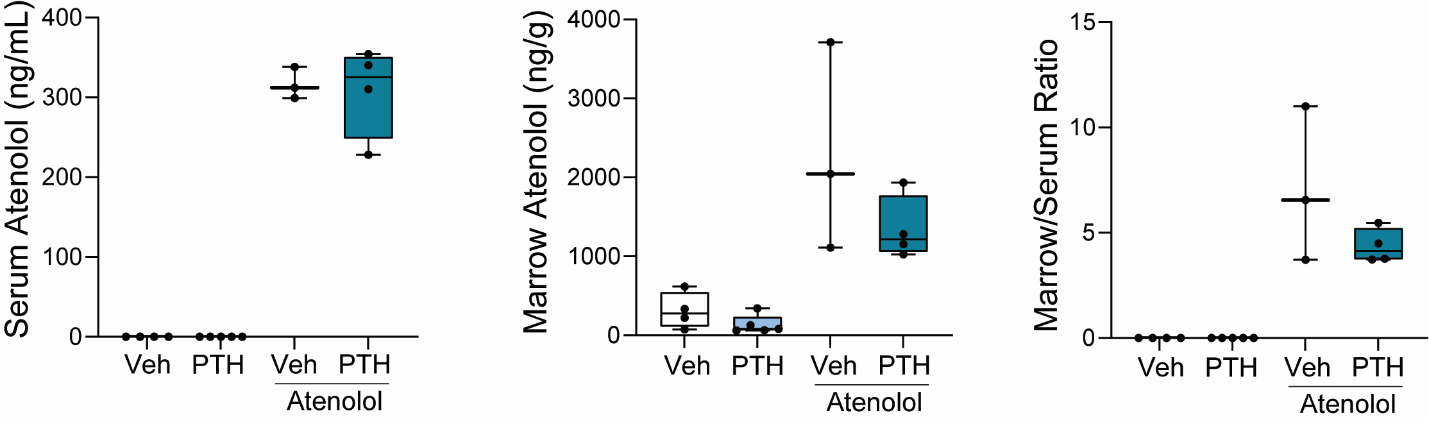
Supplementary Figure 1. Atenolol is present in the serum and marrow.** Mice were treated for 4 weeks with vehicle (white), 80 μg/kg PTH (light blue), 10 mg/kg atenolol, or PTH and atenolol (teal) for 4 weeks from 16-20 weeks of age. LC-MS/MS was used to determine atenolol concentration in the serum and marrow 1 hour after treatment. N=3-4/group. Individual points are plotted as closed circles. Boxes represent the 25^th^ to 75^th^ percentiles, with the horizontal line indicating the median.

**Supplementary Table 1. Atenolol marrow and serum concentration after single dose.**

| ID | Group | Serum Concentration (nM) | Marrow Concentration (nM) | Marrow/Serum Ratio |
| --- | --- | --- | --- | --- |
| 1 | Atenolol (30 minute) | 414 | 2234 | 5.40 |
| 2 | Atenolol (30 minute) | 349 | 1342 | 3.84 |
| 3 | Atenolol (30 minute) | 334 | 1760 | 5.28 |
| 4 | Atenolol (1 hour) | 284 | 1933 | 6.80 |
| 5 | Atenolol (1 hour) | 642 | 3230 | 5.03 |
| 6 | Atenolol (1 hour) | 705 | 3438 | 4.88 |
| 10 | Atenolol (2 hour) | 398 | 2633 | 6.62 |
| 11 | Atenolol (2 hour) | 341 | 3149 | 9.23 |
| 12 | Atenolol (2 hour) | 292 | 2400 | 8.23 |
| 7 | Vehicle (1 hour) | BLOQ (<1.00 nM) | BLOQ (<2.00 nM) | N/A |
| 8 | Vehicle (1 hour) | BLOQ (<1.00 nM) | BLOQ (<2.00 nM) | N/A |
| 9 | Vehicle (1 hour) | BLOQ (<1.00 nM) | BLOQ (<2.00 nM) | N/A |
| *Abbreviations:* BLOQ = Below Level of Quantification; M/P Ratio = Marrow:Serum Ratio. | | | | |

**Supplementary Table 2. Baseline body composition and areal bone parameters.**

|  |  |  |  |  | **2-way ANOVA *p*-values** | | |
| --- | --- | --- | --- | --- | --- | --- | --- |
|  | **Vehicle  (N=7)** | **PTH  (N=8)** | **Atenolol  (N=7)** | **PTH + Atenolol  (N=8)** | **PTH** | **Atenolol** | **Interaction** |
| **Body Mass (g)** | 20.8 ± 0.5 | 22 ± 1.6 | 22.8 ± 1.5 | 22.4 ± 1.5 | 0.5005 | **0.0249** | 0.1315 |
| **Fat-Free Mass (g)** | 17.7 ± 0.7 | 18.2 ± 1.2 | 18.9 ± 0.9 | 18.3 ± 0.9 | 0.9630 | 0.0635 | 0.1117 |
| **% Fat-Free Mass** | 84.8 ± 1.7 | 83.1 ± 1.7 | 83.0 ± 2.1 | 82.1 ± 2.4 | 0.1086 | 0.0643 | 0.5979 |
| **Fat Mass (g)** | 1.9 ± 0.3 | 2.4 ± 0.6 | 2.6 ± 0.8 | 2.7 ± 0.8 | 0.2127 | 0.0780 | 0.3770 |
| **% Fat Mass** | 9.2 ± 1.3 | 11.0 ± 2.1 | 11.1 ± 3.0 | 11.8 ± 2.8 | 0.1727 | 0.1405 | 0.5171 |
| **Total aBMD (g/cm^2^)** | 0.050 ± 0.002 | 0.050 ± 0.001 | 0.050 ± 0.001 | 0.051 ± 0.001 | 0.1723 | 0.6989 | 0.7833 |
| **Femur aBMD (g/cm^2^)** | 0.063 ± 0.002 | 0.065 ± 0.004 | 0.064 ± 0.002 | 0.066 ± 0.003 | 0.0885 | 0.4972 | 0.8402 |
| **Total aBMC (g)** | 0.43 ± 0.02 | 0.44 ± 0.02 | 0.43 ± 0.02 | 0.44 ± 0.02 | 0.2091 | 0.3841 | 0.8651 |
| Data presented as mean ± standard deviation. Abbreviations: PTH, parathyroid hormone; ANOVA, analysis of variance; aBMD, areal bone mineral density; aBMC, areal bone mineral content. | | | | | | | |

**Supplementary Table 3. Body composition and areal bone parameters of mice treated with PTH and/or atenolol.**

|  |  |  |  |  | **2-way ANOVA *p*-values** | | |
| --- | --- | --- | --- | --- | --- | --- | --- |
|  | **Vehicle  (N=7)** | **PTH  (N=8)** | **Atenolol  (N=7)** | **PTH + Atenolol  (N=8)** | **PTH** | **Atenolol** | **Interaction** |
| **Body Mass (g)** | 21.5 ± 0.7 | 22.7 ± 1.3 | 23.7 ± 1.6 | 22.9 ± 1.4 | 0.7018 | **0.0186** | 0.0519 |
| **Fat-Free Mass (g)** | 18.5 ± 0.7 | 19.0 ± 1.0 | 19.7 ± 0.9 | 19.1 ± 1.0 | 0.8141 | 0.0653 | 0.1083 |
| **% Fat-Free Mass** | 86.0 ± 0.8 | 83.8 ± 1.4 | 83.3 ± 2.5 |  | 0.0821 | 0.0201 | 0.0859 |
| **Fat Mass (g)** | 2.0 ± 0.3 | 2.3 ± 0.5 | 2.8 ± 1.0 | 2.5 ± 0.6 | 0.9969 | **0.0343** | 0.174 |
| **% Fat Mass** | 9.3 ± 1.3 | 10.2 ± 1.6 | 11.7 ± 3.4 | 10.9 ± 2.0 | 0.9877 | 0.0615 | 0.2897 |
| **Total aBMD (g/cm^2^)** | 0.052 ± 0.001 | 0.054 ± 0.001 | 0.052 ± 0.001 | 0.054 ± 0.001 | **<0.0001** | 0.6997 | 0.4569 |
| **Femur aBMD (g/cm^2^)** | 0.067 ± 0.004 | 0.071 ± 0.004 | 0.068 ± 0.004 | 0.071 ± 0.003 | **0.0128** | 0.7245 | 0.4627 |
| **Total aBMC (g)** | 0.45 ± 0.02 | 0.50 ± 0.02 | 0.47 ± 0.03 | 0.49 ± 0.02 | **0.0009** | 0.4674 | 0.3129 |
| Data presented as mean ± standard deviation. Abbreviations: PTH, parathyroid hormone; ANOVA, analysis of variance; aBMD, areal bone mineral density; aBMC, areal bone mineral content. | | | | | | | |

**Supplementary Table 4. qPCR primer information.**

| **Target Gene** | **Source/Supplier** | **Sequence** | **Catalog Number** |
| --- | --- | --- | --- |
| *Hprt* | IDT  (Coralvile, IA) | Forward: 5′-AAG CCT AAG ATG AGC GCA AG-3’  Reverse: 5 -TTA CTA GGC AGA TGG CCA CA-3’ | N/A |
| *Ctsk* | Qiagen  (Germantown, MD) | Not provided | 330001 PPM05123C |
| *Acp5* | Qiagen  (Germantown, MD) | Not provided | 330001 PPM29328F |
| *Bglap (Osteocalcin)* | IDT  (Coralvile, IA) | Forward: 5'-ACG GTA TCA CTA TTT AGG ACC TGT-3' Reverse: 5'-ACT TTA TTT TGG AGC TGC TGT GAC-3' | N/A |
| *Runx2* | IDT  (Coralvile, IA) | Forward: 5'-GAC AGA AGC TTG ATG ACT CTA AAC C-3'  Reverse: 5'-TCT GTA ATC TGA CTC TGT CCT TGT G-3' | N/A |
| *Tnfsf11 (Rankl)* | Qiagen  (Germantown, MD) | Not provided | 330001 PPM03047F |
| *Tnfrsf11b (Opg)* | Qiagen  (Germantown, MD) | Not provided | 330001 PPM03404F |
| *Adrb1* | Qiagen  (Germantown, MD) | Not provided | 330001 PPM05035A |
| *Adrb2* | Qiagen  (Germantown, MD) | Not provided | 33001  PPM04265C |
